# To Exercise at High Intensity Heart Rate or Not to Exercise at High Intensity Heart Rate?

**DOI:** 10.1002/mdc3.70499

**Published:** 2026-01-09

**Authors:** Daniel M. Corcos, Guillaume Lamotte, Nijee S. Luthra, Kathleen E. McKee

**Affiliations:** ^1^ Department of Physical Therapy & Human Movement Sciences Northwestern University Chicago IL USA; ^2^ Department of Neurology University of Utah Salt Lake City UT USA; ^3^ Department of Neurology University of California San Francisco San Francisco CA USA; ^4^ Parkinson's Elevated, Intermountain Health Salt Lake City UT USA

**Keywords:** aerobic exercise, disease modification, disease progression, over exercise, Parkinson's disease

In a letter entitled: “Running into Trouble: The Problem of Overexercise (in some),”^1^ Boon and colleagues made three points. First, they are “beginning to encounter patients who tend to overexercise with detrimental effects.” Second, clinicians should include an enquiry about the exact exercise regimen in their follow‐up routine in patients with Parkinson's disease (PD). Third, if exercise does indeed have a positive effect on the nigrostriatal system, moderate exercise might be sufficient.^2^ We completely agree with the first two points and have a different perspective on point three.

We too have encountered patients who overexercise to their detriment. But to be clear: at the population level, under‐exercise remains a far greater problem than over‐exercise. All patients should be counseled that exercise has clear benefits, that there is a recommended prescription for people with PD,^3^ more exercise is not always better, and can be detrimental. This is no different to counseling our patients that levodopa is beneficial but too much and too often is not; or that that there is a sweet spot for voltage in programming deep brain stimulation with higher voltage not always better. It is also the case that aerobic training for many hours a week can predispose some people to atrial fibrillation and atrial flutter.^4^ As such clinicians should enquire about the exercise habits of their patients and stress the importance of rest, sleep, diet and listening to their body if there is evidence of overexertion. In cases of overexertion, patients should cut back.

With respect to point three, recommendations should be derived from studies on people with PD,^3^ supported by solid preclinical data^5^ and not just from one study on mice.^2^ The literature is clear that if a person with PD wants to slow disease progression, the only studied way to do this is to exercise three times a week at 80–85% of peak heart rate.^3^ While it is true that moderate exercise is beneficial, and that overexercising is unhealthy, not informing people with PD of the benefits of high intensity exercise does them a disservice and is contrary to evidence‐based practice. It is also the case that moderate exercise in the cited mouse study exceeds the duration used in nearly all human studies.

In conclusion, patients who are able should be encouraged to exercise at high heart rate for 30 minutes 3 times a week. Clinicians should enquire about the exact exercise regimen in their follow‐up routine in PD patients. When patients are overexercising or not responding well to exercise, they should be referred to a neuro physical therapist who can help them titrate their exercise for optimum benefit. But patients must not be led to think moderate exercise is just as good as higher intensity exercise. In our clinics, we make it clear to our patients that any exercise is better than none—but if their treatment goal is to slow down their disease—the literature suggest that they exercise at a high heart rate as part of their overall Parkinson's Exercise Prescription.

## Disclosures

**Ethical Compliance Statement:** This is a letter and institutional review board, or ethics committee approval is not required. “Informed patient consent was not necessary for this work.” All four authors confirm that we have read the Journal's position on issues involved in ethical publication and affirm that this work is consistent with those guidelines.

**Funding Sources and Conflict of Interest:** The preparation of this letter was supported by the National Institute of Neurological Disorders and Stroke of the National Institutes of Health under Award Numbers U01NS113851 and K23NS123506. Research is also supported by the National Institutes of Health's National Center for Advancing Translational Sciences, Grant Number UL1TR001422. It is also supported by a generous philanthropic gift from the JCS Family Foundation to support Parkinson's Elevated. The content is solely the responsibility of the authors and does not necessarily represent the official views of the National Institutes of Health. The authors declare that there are no relevant conflicts of interest to report with respect to the writing of this letter.

**Financial Disclosures for the previous 12 months:** The authors declare that there are no additional disclosures to report.

## Author Roles

(1) Research Project: A. Conception, B. Organization, C. Execution; (2) Statistical Analysis: A. Design, B. Execution, C. Review and Critique; (3) Manuscript Preparation: A. Writing of the First Draft, B. Review and Critique.

D.M.C.: 1A, 1B, 1C, 3A, 3B.

G.L.: 3B

N.S.L.: 3B

K.E.M.: 3B

## Financial Disclosures and Conflicts of Interest

Author disclosures are available in the Supporting Information.

## Supporting information


**Data S1.** Supporting Information.

## Data Availability

Data sharing not applicable to this article as no datasets were generated or analyzed during the current study.
